# A Validated Model for Individualized Prediction of Live Birth in Patients With Adenomyosis Undergoing Frozen–Thawed Embryo Transfer

**DOI:** 10.3389/fendo.2022.902083

**Published:** 2022-05-24

**Authors:** Yaoqiu Wu, Rong Yang, Haiyan Lin, Chunwei Cao, Xuedan Jiao, Qingxue Zhang

**Affiliations:** ^1^ Guangdong Provincial Key Laboratory of Malignant Tumor Epigenetics and Gene Regulation, Sun Yat-Sen Memorial Hospital, Sun Yat-Sen University, Guangzhou, China; ^2^ Reproductive Medicine Center, Sun Yat-Sen Memorial Hospital, Sun Yat-Sen University, Guangzhou, China

**Keywords:** adenomyosis, uterine size, live birth, GnRH-a, prediction model

## Abstract

**Purpose:**

This study aimed to develop a predictive tool for live birth in women with adenomyosis undergoing *in vitro* fertilization (IVF)/intracytoplasmic sperm injection (ICSI) treatment.

**Methods:**

A total of 424 patients with adenomyosis who underwent frozen–thawed embryo transfer (FET) from January 2013 to December 2019 at a public university hospital were included. The patients were randomly divided into training (*n* = 265) and validation (*n* = 159) samples for the building and testing of the nomogram, respectively. Multivariate logistic regression (MLR) was developed on the basis of clinical covariates assessed for their association with live birth.

**Results:**

In total, 183 (43.16%) patients became pregnant, and 114 (26.88%) had a live birth. The MLR showed that the probability of live birth was significantly correlated with age [odds ratio (OR), 3.465; 95% confidence interval (CI), 1.215–9.885, *P* = 0.020], uterine volume (OR, 8.141; 95% CI, 2.170–10.542; *P* = 0.002), blastocyst transfer (OR, 3.231; 95% CI, 1.065–8.819, *P* = 0.023), twin pregnancy (OR, 0.328; 95% CI, 0.104–0.344, *P* = 0.005), and protocol in FET (*P* < 0.001). The statistical nomogram was built based on age, uterine volume, twin pregnancy, stage of the transferred embryo, and protocol of FET, with an area under the curve (AUC) of 0.837 (95% CI: 0.741–0.910) for the training cohort. The AUC for the validation cohort was 0.737 (95% CI: 0.661–0.813), presenting a well-pleasing goodness-of-fit and stability in this model.

**Conclusions:**

This visual and easily applied nomogram built on the risk factors of live birth in patients with adenomyosis provides useful and precise information for physicians on individualized decision-making during the IVF/ICSI procedure.

## Background

Adenomyosis is a common gynecological disorder where endometrial glands and stroma surrounded by hyperplastic smooth muscle were found within the myometrium (1, 2). It affects up to 24.4% of infertile women, which represents a clinical issue associated with pelvic pain, excessive vaginal bleeding, enlarged uterus, and infertility (3, 4). Adenomyosis has been reported to adversely impact fertility *via* abnormal uterine contractility, including altered endometrial function and receptivity, and impaired implantation (5). Besides this, a patient with adenomyosis was also linked with poor obstetrical outcomes, including preeclampsia, placental malposition, preterm delivery, and preterm premature rupture of membrane (6–8).

Intracytoplasmic sperm injection (ICSI) and *in vitro* fertilization (IVF) are extensively being used for managing adenomyosis-related infertility. However, the results varied from one report to another, with some showing identical outcome as in patients without adenomyosis and others presenting increased miscarriage and lower clinical pregnancy rates (1). Several studies provided evidence that an enlarged uterus in adenomyosis had an adverse impact on pregnancy outcomes by the morphological and functional pathological changes (9, 10). In addition, results from some trials on adenomyosis showed that applying gonadotrophin-releasing hormone agonists (GnRH-a) before IVF/ICSI cycle exerts positive effects on increasing the clinical pregnancy rate in patients with adenomyosis (11–13). The success for a patient with adenomyosis-associated infertility in IVF/ICSI procedure to achieve pregnancy depends on a number of factors. Although several scoring systems have been published to evaluate the pregnancy rate after IVF/ICSI in infertile patients, the current guidelines are based on general rather than individual clinic data. Despite the availability of these models, few of them are applicable for patients with adenomyosis and cannot evaluate the chances of live birth for individual adenomyosis patient. In this aspect, setting up of a predictive calculation model in live birth with a combination of all the risk factors in patients with adenomyosis will be beneficial for the medical staff in the decision-making process and promoting adherence to medication through risk-informed counselling.

The aim of the current study was, therefore, to develop a nomogram model on retrospective data analysis to predict the probability of live birth for patients with adenomyosis.

## Patients and Methods

### Data Sources

Women diagnosed with adenomyosis and undergoing frozen–thawed embryo transfer during 2013 and 2019 at Sun Yat-Sen Memorial Hospital, Guangzhou, China, were screened for this retrospective cohort study. The patients were included if they had received their first frozen–thawed embryo transfer (FET) cycle with autologous embryos. The exclusion criteria included congenital uterine malformation (unicornuate, bicornuate, and septate uterus), intrauterine adhesion, uterine malformation, and leiomyoma. Couples who received a preimplantation genetic screening or underwent preimplantation genetic diagnosis were excluded. The indications for IVF/ICSI included the tubal factor, male factor, and immunity factor. Demographic data on age, body mass index (BMI), infertility duration, basal sexual hormone levels (tested on days 2 to 3 of the menstrual cycle), uterine volume prior to embryo transfer (ET) (long diameter × width diameter ×anteroposterior diameter × *π*/6) (14), type of adenomyosis (diffusion or focal), endometrial thickness, and protocol of FET were obtained from the clinical database.

The diagnosis of adenomyosis was ascertained by a detailed chart review, including visit notes and ultrasound and operative reports as well as pathology reports. The diagnosis was defined with two or more transvaginal sonographic criteria that included heterogeneous myometrial area, globular asymmetric uterus, irregular cystic spaces, myometrial linear striations, poor definition of the endometrial myometrial junction, myometrial anterior posterior asymmetry, thickening of the anterior and posterior myometrial wall, and increased or decreased echogenicity (15, 16). All identified adenomyosis cases were confirmed by two experienced sonographers. Diffuse adenomyosis was defined as an outer myometrium extensive disease with the endometrial glands and stroma scattered throughout the uterine musculature, and focal adenomyosis included adenomyoma, defined as grossly circumscribed adenomyotic masses within the myometrium (9, 17).

### Frozen–Thawed Embryo Transfer Procedure

FET was performed through a natural cycle (NC) or through hormone replacement therapy (HRT) cycles with endometrial preparation by exogenous estrogen and progesterone or through the cycle adding gonadotrophin-releasing hormone agonists (GnRH-a) before estradiol. Among the patients with GnRH agonist pre-treatment, long-acting GnRH-a was administrated with up to three injections of 3.75 mg of triptorelin acetate (Ipsen Pharma Biotech, France) (18). No more than two embryos were transferred. The luteal-supported phase was administered by the vaginal administration of micronized progesterone (400 mg/day). Pregnancies were diagnosed by an increasing concentration of serum β-hCG, which was tested 14 days after the embryo transfer (18). Clinical pregnancies were confirmed by the presence of the gestational sac on vaginal ultrasound examination during the fifth week. Twin pregnancy was confirmed by ultrasound examination during the 12th week. A live birth is defined as any live baby born after the 24th week of pregnancy.

### Data Analysis

Statistics with Gaussian distribution were presented as mean ± SD, and categorical variables were described as absolute frequencies (**Table 1**). Youden Index was used to determine the optimal cutoff point of the uterine volume associated with live birth. External validation was chosen in the study so that the patients enrolled were divided into a training set (*n* = 265) and a validation set (*n* = 159) by the sampling techniques of random numbers. Statistical analyses were performed using the STATA 14.0 MP software and Regression Modeling Strategies (R version 3.6.3). To build up the nomogram and measure the area under the curve (AUC), we used the “regplot”, “pROC”, and “rms” in R software (19). Differences between groups were compared using Student’s *t*-test or chi‐square test as appropriate.

**Table 1:** Characteristics of the patients with and without live birth.

Characteristics^a^	Without live birth	With live birth	*P*
	*n* = 310	*n* = 114	
Age, years	34.94 ± 4.68	31.92 ± 3.95	<0.001*
Infertility duration, years	4.53 ± 3.79	4.20 ± 3.05	0.403
BMI, kg/m^2^	21.58 ± 2.93	20.55 ± 2.20	0.001*
AMH, IU/L	3.22 ± 3.12	3.74 ± 2.88	0.337
FSH, IU/L	8.65 ± 3.77	7.98 ± 2.13	0.087
LH, IU/L	5.39 ± 2.48	5.60 ± 2.65	0.422
E2, pg/ml	50.29 ± 15.28	50.60 ± 36.60	0.968
T, ng/ml	1.24 ± 0.82	1.69 ± 4.90	0.150
Type of adenomyosis, *n* (%)			0.721
Diffuse	173 (65.29)	103 (64.78)	
Focal	92 (34.71)	59 (35.22)	
Uterine diameters prior to ET			
Width diameter, cm	5.51 ± 1.17	5.11 ± 0.99	0.001*
Anteroposterior diameter, cm	5.34 ± 1.17	4.97 ± 0.86	0.002*
Long diameter, cm	5.51 ± 1.07	5.51 ± 0.99	0.001*
Uterine volume, cm^3^	91.13 ± 43.44	71.60 ± 26.53	0.000*
Stage of embryo transfer			0.165
Cleavage, *n* (%)	50 (16.13)	25 (21.93)	
Blastocyst, *n* (%)	260 (83.87)	89 (78.07)	
Protocol of FET			0.050
HRT	149 (48.06)	24 (21.05)	
GnRHa-HRT	127 (40.97)	53 (46.49)	
NC	34 (10.97)	37 (32.46)	
Endometrial thickness (mm)	10.04 ± 2.56	9.21 ± 3.00	0.128
Pregnancy type			
Singleton pregnancy, *n* (%)		83 (72.81)	
Twin pregnancy, *n* (%)		31 (27.19)	

BMI, body mass index; AMH, anti-Mullerian hormone; FSH, follicle-stimulating hormone; E2, estrogen; T, testosterone; ET, embryo transfer; HRT, hormone replacement therapy; NC, nature cycle.

a Continuous variables are expressed as mean ± SD and categorical variables as absolute frequencies, n (%).

*P < 0.05 was considered statistically significant.

### Development and Validation of the Model

The training cohort of 265 patients was used to develop the nomogram for predicting the patient-specific probability of live birth in women with adenomyosis. The end-point of the study was live birth rate after FET cycles. Backward variable selection was performed to determine independent covariates. Logistic regression model was used for multivariate analysis, including the univariate analysis of significant variables (*P* < 0.05) (**Supplementary Table S1**). The coefficient for each independent covariate and the constant were generated in the equation by MLR analysis (20). The variables entered into the nomogram model were age, uterine volume, stage of the transferred embryo, twin pregnancy, and protocol of FET in the study. The values for each of the model covariates were mapped to points on a scale ranging from 0 to 100, and the total points obtained for each model corresponded to the probability of a live birth (19).

The model was applied to data from a sample of 159 patients (validation set) for external validation with a bootstrapping technique to obtain relatively unbiased estimates (1,000 repetitions). The bootstrapping method is based on resampling obtained by randomly drawing data and replacing them with samples from the original dataset (21). The predictive accuracy of the models was measured using the average optimism of the AUC. A precise prediction model would result in a plot where the observed and predicted probabilities fall along the diagonal (19).

## Results

### Description of the Study Population

A total of 424 patients with adenomyosis who underwent frozen–thawed embryo transfer from January 2011 to December 2019 were identified as eligible and were analyzed in this study. In total, 183 (43.16%) patients became pregnant, and 114 (26.88%) had a live birth. The patients with live birth were younger (31.92 ± 3.95 *vs*. 34.94 ± 4.68, *P* < 0.001), slimmer (20.55 ± 2.20 *vs*. 21.58 ± 2.93, *P* = 0.001), and had a smaller uterine size (71.60 ± 26.53 *vs*. 91.13 ± 43.44, *P* < 0.001) compared with those patients without a live birth (**Table 1**). The blastocyst stage and GnRHa-HRT protocol for FET increased the live birth rate in patients with adenomyosis (*P* < 0.001) (**Table 1**). The patients were divided into a training set and a validation set by the sampling techniques of random numbers. The model was built from a training cohort of 265 patients and was validated on an independent validation cohort of 159 patients. **Table 2** summarizes the epidemiological, clinical, biostatistical, and treatment strategies for the training and validation cohorts. No significant difference was observed in the patients’ characteristics between the two cohorts. A total of 79 patients (29.81%) in the training cohort had a live birth, while 35 patients (22.01%) in the validation cohort had a live birth.

**Table 2:** Patient characteristics in the training and the validation cohorts.

Characteristics^a^	Training set	Validating set	*P*
	*n* = 265	*n* = 159	
Live birth, *n* (%)	79 (29.81)	35 (22.01)	0.080
Age, years	34.05 ± 4.79	34.26 ± 4.52	0.652
Infertility duration, Years	4.27 ± 3.41	4.57 ± 3.91	0.196
BMI, kg/m^2^	21.41 ± 2.82	21.14 ± 2.74	0.331
AMH, IU/L	3.07 ± 2.83	3.76 ± 3.37	0.195
FSH, IU/L	8.65 ± 3.77	7.98 ± 2.13	0.087
LH, IU/L	5.27 ± 2.68	5.34 ± 2.87	0.822
E2, pg/ml	53.33 ± 15.78	46.60 ± 12.07	0.285
T, ng/ml	0.43 ± 0.24	0.46 ± 0.26	0.723
Type of adenomyosis, *n* (%)			0.721
Diffuse	173 (65.29)	103 (64.78)	
Focal	92 (34.71)	59 (35.22)	
Uterine diameters prior to ET			
Width diameter, cm	5.41 ± 1.14	5.38 ± 1.13	0.415
Anteroposterior diameter, cm	5.21 ± 1.11	5.30 ± 1.11	0.808
Long diameter, cm	5.56 ± 1.05	5.67 ± 1.09	0.300
Uterine volume, cm^3^	84.81 ± 40.66	87.66 ± 40.35	0.485
Stage of embryo transfer			0.107
Cleavage, *n* (%)	53 (20.00)	25 (13.84)	
Blastocyst, *n* (%)	212 (80.00)	89 (86.16)	
Protocol of FET			0.219
HRT	115 (43.40)	58 (36.48)	
GnRHa-HRT	104 (39.25)	76 (47.80)	
NC	46 (17.35)	25 (15.72)	
Endometrial thickness (mm)	9.87 ± 2.72	9.91 ± 2.62	0.889
Pregnancy type			0.193
No pregnancy	142 (53.58)	99 (62.26)	
Singleton pregnancy, n (%)	71 (26.79)	37 (23.27)	
Twin pregnancy, n (%)	52 (19.62)	23 (14.47)	

BMI, body mass index; AMH, anti-Mullerian hormone; FSH, follicle-stimulating hormone; E2, estrogen; T, testosterone; ET, embryo transfer; HRT, hormone replacement therapy; NC, nature cycle.

a Continuous variables are expressed as mean ± SD and categorical variables as absolute frequencies, n (%).

*P < 0.05 was considered statistically significant.

The logistic regression analysis revealed that blastocyst transfer, small uterine size, and GnRH-a pretreatment prior to FET improved the live birth, but twin pregnancy negatively impacted the live birth.

**Figure 1:**
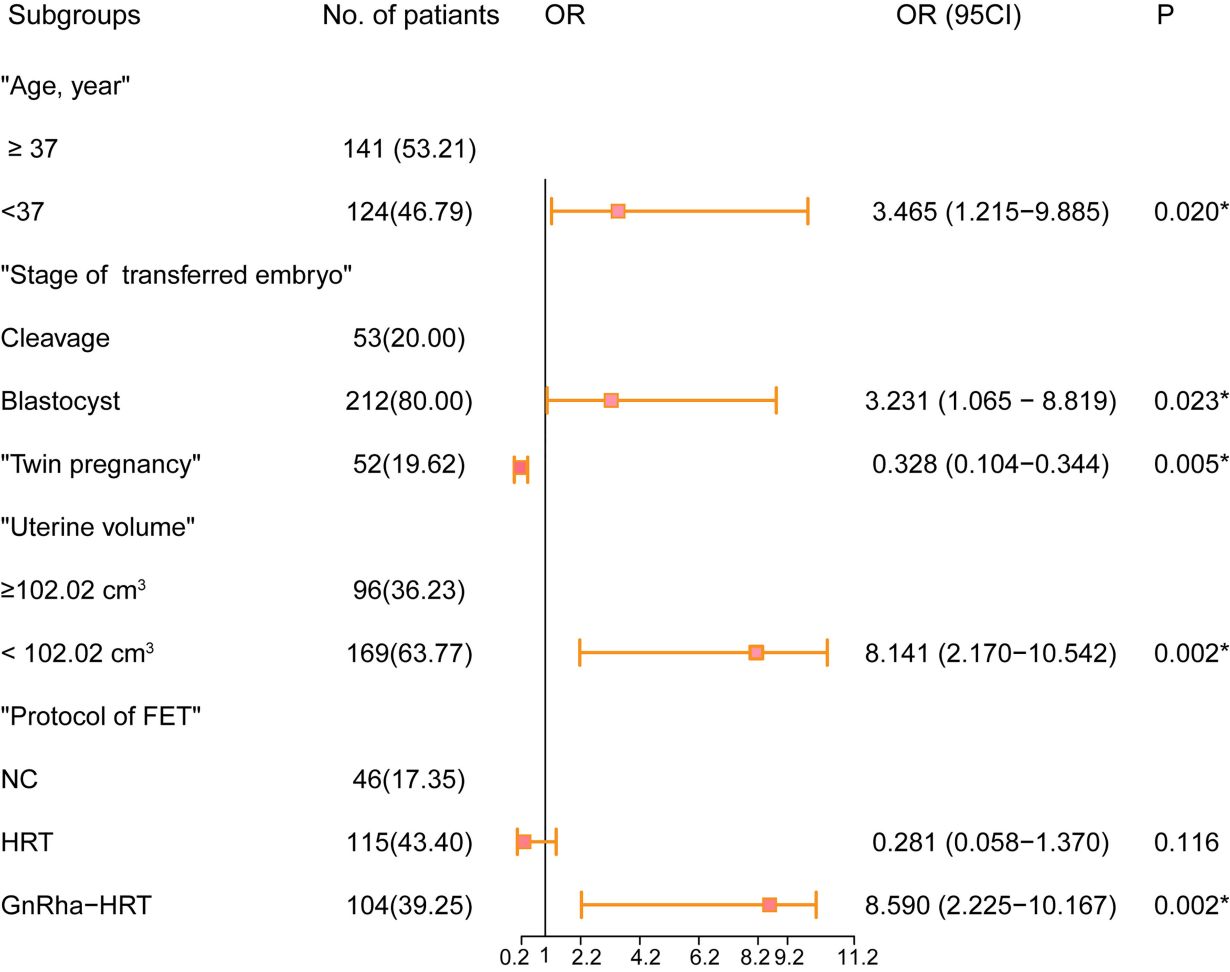
Forest plot of the predictive factors of live birth in the multivariable analysis of the training cohort. OR and 95% CI are presented to show the risk of predictive factors. FET, frozen–thawed embryo transfer; OR, odds ratio; CI, confidence interval. *P < 0.05 was considered statistically significant.

The optimal cutoff point of the uterine volume prior to ET related to live birth was 102.02 cm^3^ (AUC = 0.603, *P* = 0.003) according to the Youden Index. **Supplementary Table S1** summarizes the univariable and multivariable analyses. The univariate logistic regression analysis showed that the live birth rate was significantly correlated with age (*P* = 0.018), uterine volume prior to ET <102.02 cm^3^ (*P* < 0.001), twin pregnancy (*P* < 0.001), stage of the transferred embryo (*P* = 0.012), and protocol in FET (*P* < 0.001). In the MLR analysis of the training cohort, the probability of a live birth was significantly correlated with the age <37 years old [odds ratio (OR), 3.465; 95% CI, 1.215–9.885, *P* = 0.020], uterine volume prior to ET <102.02 cm^3^ (OR, 8.141; 95% CI, 2.170–10.542; *P* = 0.002), blastocyst transfer (OR, 3.231; 95% CI, 1.065–8.819, *P* = 0.023), twin pregnancy (OR, 0.328; 95% CI, 0.104–0.344, *P* = 0.005), and protocol in FET (*P* < 0.001) (**Figure 1**). Blastocyst transfer, small uterine size, and GnRH-a pretreatment were associated with an increased probability of live birth, but twin pregnancy decreased the probability.

### Development of the Models From the Training Cohort

**Figure 2:**
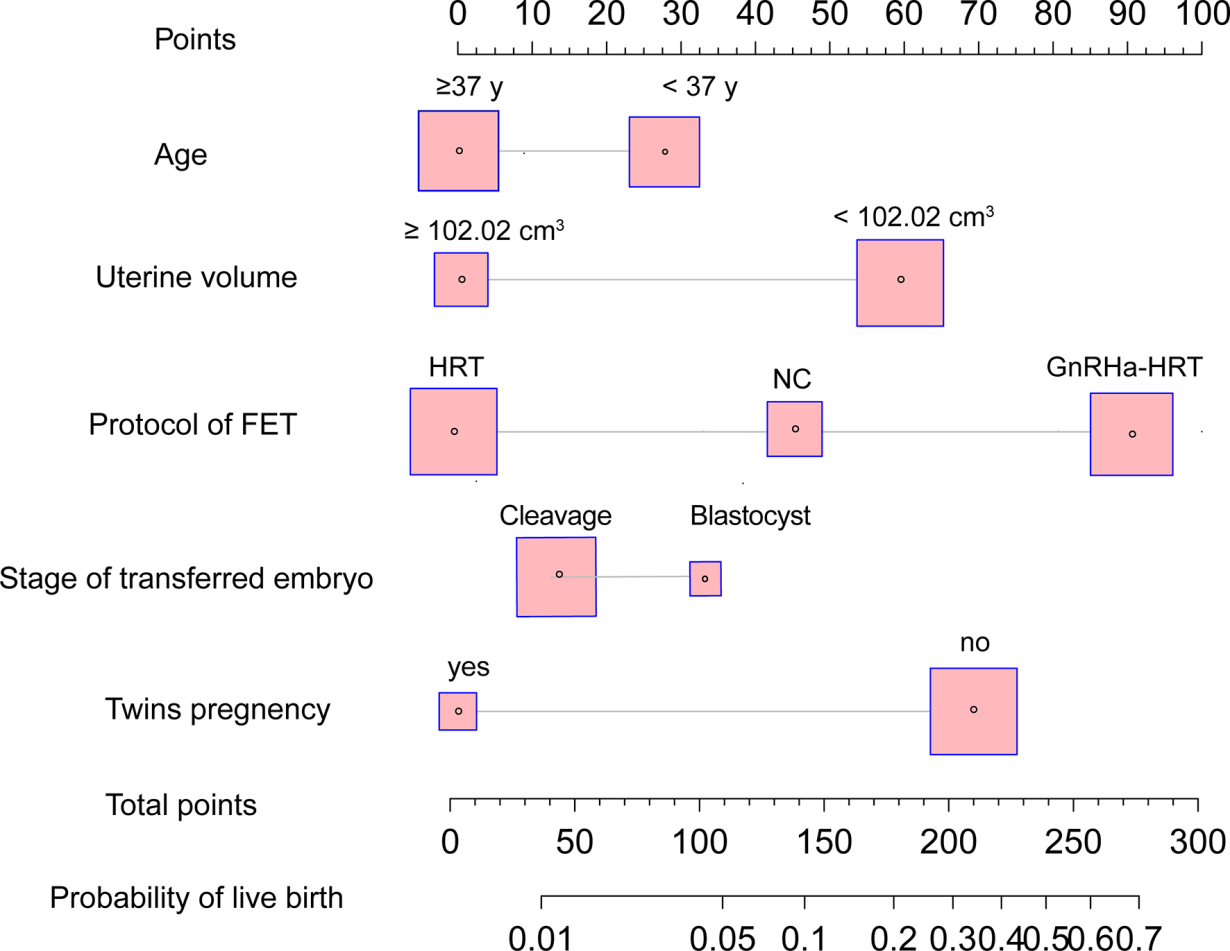
Nomogram to predict the probability of live birth in adenomyosis-related infertility patients undergoing FET. The probability of a live birth is calculated by drawing a line to the point on the axis for each of the following variables: stage of transferred embryo, age, twin pregnancy, protocol of FET, and uterine volume prior to ET. The points for each variable are summed up and located on the total points line. Next, a vertical line is projected from the total points line to the predicted probability bottom scale to obtain the individual probability of a live birth. FET, frozen–thawed embryo transfer.

Based on the univariable and multivariable logistic regression analyses that we performed, a nomogram incorporating the significant risk factors was established to predict the probability of a live birth (**Figure 2**). Total scores were calculated based on age, stage of the transferred embryo, uterine volume, twin pregnancy, and protocol of FET. The equation describing the probability of a live birth was as follows: *P* = 1/[1 + exp (-X)], where *X* = 0.4755302 + 0.1091108 × *V1* + 0.0882141 × *V2* - 0.3309371 × *V3* + 0.1281561 × *V4* + 0.2339871, where *V1* was age (1 if <37 years old and 0 if ≥37 years old), *V2* was blastocyst transfer (0 if no and 1 if yes), *V3* was twin pregnancy (1 if no and 0 if yes), *V4* was the protocol of FET (2 if GnRH-a HRT, 1 if NC, and 0 if HRT), and *V5* was uterine volume (1 if <102.02 cm^3^ and 0 if ≥102.02 cm^3^). The nomogram derived from this equation is reported in **Figure 2**.

### Validation of Predictive Accuracy

**Figure 3:**
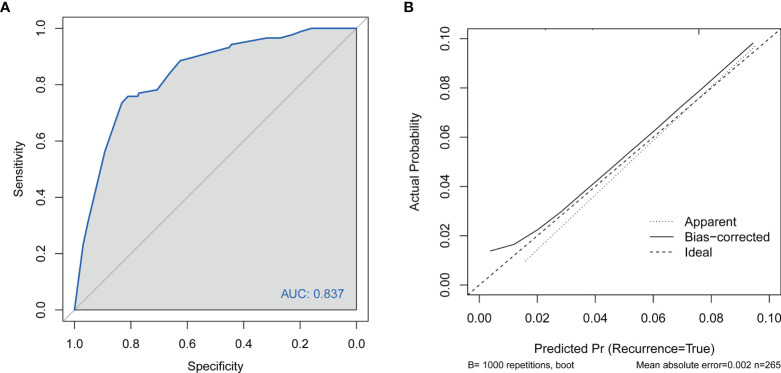
**(A)** Discrimination for the training cohort. ROC curve of the model with an area under the curve of 0.837 (95% confidence interval: 0.741–0.910). **(B)** Calibration of the nomogram to predict live birth in patients with adenomyosis undergoing FET. FET, frozen–thawed embryo transfer; CI, cervical insufficiency; ROC, receiver operating characteristic curve.

No significant difference was observed between the predicted probability obtained from the bootstrap correction and the actual probabilities of live birth (*P* = 0.186), which implied that the nomogram was well calibrated. The model demonstrated an AUC of 0.837 (95% confidence interval: 0.741–0.910) in the training cohort (**Figures 3A, B**), which denoted good performance. The AUC of the receiver operating characteristic (ROC) curve in the validation set was 0.737 (95% confidence interval: 0.661–0.813), which indicated fair performance.

## Discussion

On the basis of 424 infertile patients with adenomyosis who underwent FET, we have first created a predictive nomogram tailored to the individual patient and capable of reliably generating the numerical probabilities of live birth. The nomogram was developed in a training cohort including 265 patients and tested on an external independent validation cohort including 159 patients. Both calibration and discrimination were used to evaluate the performance. This graphical tool is a simple and straightforward calculator, integrating five predictive variables, that was easily accessible during the assisted reproductive technology (ART) treatment constituting of age, uterine volume, protocol of FET, type of pregnancy, and stage of the transferred embryo. Moreover, this model firstly integrated the potential risks in fetal loss of a patient with adenomyosis into one graphical calculator, which is of particular interest for clinicians to make an informed decision on the timing and protocol of FET, stage, and number of embryos to transfer.

An enlarged uterus in patients negatively impacted the live birth rate as revealed by MRL in our study. Endometrial tissues within the myometrium induce hyperplasia and hypertrophy of the adjacent smooth muscle, resulting in uterus enlargement which is considered an important feature of adenomyosis (10). The morphological and functional pathological changes caused by hyperplasia and hypertrophy of the adjacent smooth muscle weaken the scalability and coordination of the uterus, which adversely influence the patient’s pregnancy and delivery procedure (1, 10, 22). It was suggested that adenomyosis patients with an enlarged uterus suffer from a high rate of miscarriage and preterm delivery and have babies who were small for gestational age (10, 23). In addition, Kim *et al.* indicated that preterm delivery in pregnant patients with adenomyosis can be predicted through uterine wall thickness measurement in the second trimester (22). Recently, a retrospective study from Li *et al.* demonstrated that adenomyosis patients with a larger uterine volume suffered from lower live birth rate due to a higher incidence of miscarriage (9). A prospective study by Hawkins *et al.* also revealed that women with uterine lengths longer than 9 cm were more likely to experience spontaneous abortions (24). Consistently, a uterine volume larger than 102.02 cm^3^ was associated with a lower live birth rate in our study. Therefore, the use of uterine volume as a significant determinant factor in pre-pregnancy examinations should never be ignored. Routine checks for uterine size during ART treatment are beneficial for detecting patients who were at an increased risk so that proper protocol for the subsequent FET can be chosen and preventive measures can be taken in early pregnancy.

GnRH-a pre-treatment in FET cycles significantly improved the live birth rate in our retrospective study. Consistently, several studies suggested that the administration of GnRH agonist increased the implantation rate, clinical pregnancy rate, and ongoing pregnancy rate of patients with adenomyosis in FET cycles (11, 25). The adenomyosis tissue, which contained estrogen, progesterone, and androgen receptors, develops in an estrogen-dependent manner (26). The administration of GnRH agonist can suppress the hypothalamic–pituitary axis, resulting in a hypoestrogenic status, and then suppress the proliferation of cells derived from the endometrium, reducing the size of pathologic lesions in patients with adenomyosis (27, 28). Moreover, the expression of aromatase cytochrome P450, a protein overexpressed in women with adenomyosis and that catalyzed the conversion of androgen to estrogen, can be decreased by GnRH agonist (29). Our results show that, after adjustment for confounding factors, GnRH agonist pre-treatment is associated with increased live births in patients with adenomyosis in FET cycles. With the increasing use of embryo freezing–thawing, pretreatment with GnRH agonist is recommended for adenomyosis patients in FET cycles.

A patient’s age has been considered to be a significant prognostic factor in reproductive medicine and frequently involved in assessing the probability of a live birth or pregnancy (30). Uterine adenomyosis mostly occurs in women over the age of 35 years old, and the average age of patients included in our study was up to 34, which was associated with adverse pregnancy outcomes in this study.

Consistent with previously published studies (31, 32), our study showed that the increased live birth rate was significantly associated with blastocyst transfer than cleavage embryo transfer. Besides this, twin pregnancy—a well-understood risk factor of adverse obstetric outcomes (33)—was a strong collective factor in our model. Therefore, single blastocyst embryo transfer, which is highly recommended in ET cycles for its high live birth rate, is encouraged in patients with adenomyosis, especially those with an enlarged uterus.

However, some limitations of the current study have to be highlighted. First, we could not avoid the deviation of uterine diameter measurement by different clinicians. Second, the diagnosis for adenomyosis relied on ultrasound results, such that mild adenomyosis might have been misclassified. Third, the retrospective nature of the study cannot exclude all biases. Despite these limitations, our nomogram model for predicting live birth rates could be a useful tool to help doctors and adenomyosis patients undergoing IVF/ICSI procedure decide on embryo transfer option and to give special attention during prenatal visits.

## Conclusion

In conclusion, an objective and accurate prediction nomogram model for live birth rate was drawn up and validated in infertile patients with adenomyosis. Relative risk assessment could be performed during infertility consultation, and appropriate measures could be carried out in advance to minimize the probability of fetal loss. In addition, our results support the concept that pretreatment of GnRH-a for reducing the lesion size before FET effectively increased the probability of a live birth.

## Data Availability Statement

The raw data supporting the conclusions of this article will be made available by the authors without undue reservation.

## Ethics Statement

This study was approved by the ethical standards of the Ethics Committee of The Sun Yat-Sen Memorial Hospital of China (SYSEC-KY-KS-2020-127). The patients/participants provided their written informed consent to participate in this study.

## Author Contributions

QZ supervised the entire study, including the procedures, conception, design, and completion of the study data. HL and CC revised the article. RY and XJ were responsible for the collection of data. YW contributed to data collection and analysis and drafted the article. All authors contributed to the article and approved the submitted version.

## Funding

This study was supported by the National Natural Science Foundation of China (81971332), the Natural Science Foundation of Guangdong Province (2020A1515011126), and Sun Yat-Sen University Clinical Research 5010 Program (2016004).

## Conflict of Interest

The authors declare that the research was conducted in the absence of any commercial or financial relationships that could be construed as a potential conflict of interest.

## Publisher’s Note

All claims expressed in this article are solely those of the authors and do not necessarily represent those of their affiliated organizations, or those of the publisher, the editors and the reviewers. Any product that may be evaluated in this article, or claim that may be made by its manufacturer, is not guaranteed or endorsed by the publisher.

## Supplementary Material

Supplementary Table 1 Univariate and multivariate analyses of factors predicting the live birth in patients with adenomyosis undergoing frozen–thawed embryo transfer. BMI, body mass index; AMH, anti-Mullerian hormone; HRT, *in vitro* fertilization/intracytoplasmic sperm injection; NC, nature cycle, FET, frozen–thawed embryo transfer. **P < *0.05 was considered statistically significant.

## Abbreviations

BMI, body mass index; FSH, follicle-stimulating hormone; E2, estrogen; T, testosterone; HRT, hormone replacement therapy; FET, frozen–thawed embryo transfer; IVF/ICSI, *in vitro* fertilization/intracytoplasmic sperm injection; ART, assisted reproductive technology; GnRH-a, gonadotrophin-releasing hormone agonists; hCG, human chorionic gonadotropin; LBR, live birth rate; ROC, receiver operating characteristic curves; SD, standard deviation; ORs, odds ratios; CI, confidence interval.
