# Supplementary material for: A Validated Model for Individualized Prediction of Live Birth in Patients With Adenomyosis Undergoing Frozen–Thawed Embryo Transfer
Source: Front Endocrinol (Lausanne). 2022 May 24;13:902083. doi: 10.3389/fendo.2022.902083 (PMC9171040; doi:10.3389/fendo.2022.902083)
Supplement: Supplementary Table 1 — Univariate and multivariate analyses of factors predicting the live birth in patients with adenomyosis undergoing frozen–thawed embryo transfer. BMI, body mass index; AMH, anti-Mullerian hormone; HRT, in vitro fertilization/intracytoplasmic sperm injection; NC, nature cycle, FET, frozen–thawed embryo transfer. *P < 0.05 was considered statistically significant. [file Table_1.docx]

Table S1. Univariate and multivariate analysis of factors predicting the live birth

in patients with adenomyosis undergoing frozen-thawed embryo transfer

|  | Univariate analysis | | Multivariate analysis | |
| --- | --- | --- | --- | --- |
|  | OR  95% CI | *p* value | OR  95% CI | *P* value |
| Age < 37 years | 1.842 (1.110-3.052) | 0.018* | 3.465 (1.215-9.885) | 0.020* |
| Infertility duration | 1.000 (0.932-1.078) | 0.949 |  |  |
| Uterine volume < 102.02 cm^3^ (prior ET) | 1.163  (1.072-3.370) | <0.001* | 8.141 (2.170-10.542) | 0.002* |
| AMH, IU/L | 0.798 (0.571-1.115) | 0.187 |  |  |
| BMI < 24 kg/m^2^ | 5.179 (0.815-8.984) | 0.293 |  |  |
| Type of Adenomyosis |  | 0.582 |  |  |
| Diffuse | Reference |  |  |  |
| Focal | 1.435 (0.397-5.180) |  |  |  |
| Endometrial thickness (mm) | 1.022 (0.807-1.294) | 0.856 |  |  |
| Stage of transferred embryo |  | 0.012* |  | 0.023* |
| Cleavage | Reference |  | Reference |  |
| Blastocyst | 8.978 (1.630 - 9.438) |  | 3.231 (1.065 - 8.819) |  |
| Protocol of FET |  | <0.001* |  | <0.001* |
| NC | Reference |  | Reference |  |
| HRT | 3.237 (0.464-10.866) |  | 0.281 (0.058-1.370) |  |
| GnRH-a-HRT | 4.845 (2.552-8.082) |  | 8.590 (2.225-10.167) |  |
| Twin pregnancy | 0.171 (0.093-0.318) | <0.001* | 0.328 (0.104-0.344) | 0.005* |

*Abbreviations: BMI, body mass index; AMH, anti-mullerian hormone; HRT, in vitro fertilization/ intracytoplasmic sperm injection; NC, nature cycle, FET, frozen-thawed embryo transfer; *P < 0.05 was considered statistically significant.*
